# Secondhand Suspicions: Breast Cancer and Passive Smoking

**DOI:** 10.1289/ehp.115-a136

**Published:** 2007-03

**Authors:** Kellyn S. Betts

Does a young woman living with a smoker or taking a job working in a smoky bar have a greater chance of developing breast cancer? Some scientists believe that such situations can indeed raise a woman’s risk of developing breast cancer before the age of 50. Because epidemiological and toxicological studies show that women’s breast tissue may be especially sensitive to exposure to carcinogens prior to first pregnancy, these researchers contend that public education should be directed at alerting adolescents and young women to the potential risk. However, not everyone in the international public health community agrees that the evidence to date supports a link between passive smoking and breast cancer, and some say that women are being alarmed unnecessarily. This disagreement has sparked debate that is sometimes heated.

The stakes are high because breast cancer is the most common cancer in women in industrialized countries, according to the WHO. It is the leading cancer killer of nonsmoking women, and second only to lung cancer deaths among women who smoke.

Among the researchers interviewed for this article who disagree that there is enough evidence to link secondhand smoke (SHS) with breast cancer, the majority call the evidence to date “suggestive but not sufficient,” as the Surgeon General’s 2006 report, *The Health Consequences of Involuntary Exposure to Tobacco Smoke*, put it. That characterization is based largely on the fact that the research considered when the Surgeon General’s report was being amassed did not clearly link even active smoking to breast cancer. Researchers in this camp do, however, stress that ongoing campaigns to prohibit smoking in public will protect the whole of society against the wide variety of ills proven to be caused by SHS. These include lung cancer, cardiovascular disease, and sudden infant death syndrome, among others.

A smaller group contends that the question of whether or not SHS causes breast cancer is a political issue with the potential to compromise the scientific process. “A premature decision about causality could jeopardize the credibility of the entire review process and all of the other, established effects of secondhand smoke,” says Michael Thun, national vice president of epidemiology and surveillance research for the American Cancer Society. Adds Valerie Beral, director of the University of Oxford Cancer Research UK Epidemiology Unit, “To prematurely come to conclusions about the causation when there is a big division in the scientific community . . . is bad science.”

Thun debated the subject in a series of public forums held in conjunction with scientific meetings. Taking the opposing view was Kenneth C. Johnson, a research scientist with the Public Health Agency of Canada, who was one of the first scientists to discern a potential link. During the debates, Johnson pointed out there are about the same number of studies linking breast cancer to passive smoking as there were linking lung cancer to SHS in 1986, when the Surgeon General concluded that passive smoking caused lung cancer. Johnson also says that more of the breast cancer studies are statistically significant, and that the estimated risk for breast cancer is higher.

## The Importance of Carcinogens in Tobacco Smoke

The suspicion that exposure to SHS could cause breast cancer dates back more than two decades. Among the more than 50 carcinogens in tobacco smoke are approximately 20 substances listed as mammary carcinogens by the International Agency for Research on Cancer. These include compounds such as dibenzo[*a,1*]pyrene, which the research literature identifies as an extremely potent carcinogen in mammary tissue.

The chemicals in tobacco smoke are a mixed bag of directly genotoxic DNA-damaging compounds (“initiators”), compounds that enhance the action of these initiators (“promoters”), and compounds that do both, says Andrew Salmon, a toxicologist at the California EPA’s Office of Environmental Health Hazard Assessment (Cal/EPA OEHHA). Some of these substances are more abundant in sidestream smoke, which comes off the tip of the cigarette, than the smoke inhaled by smokers themselves. This sidestream smoke is the major source of SHS.

Numerous studies have shown that toxicants from cigarette smoke reach rodent mammary tissue and can form the DNA adducts believed to represent the first step of carcinogenesis. If not repaired or if repaired incorrectly, these modifications may eventually lead to mutations and ultimately cancer. Irma Russo, a member of the Fox Chase Cancer Center’s Medical Science Division, says, “We know that [tobacco smoke] is carcinogenic to the human breast [because we have] utilized some of the carcinogens, such as benzo[*a*]pyrene, that are present in tobacco smoke and induced tumor formation in breast epithelial cells.” What is less clear, she says, is when and how this exposure causes cancer in women.

Jonathan Li, a professor in the University of Kansas Medical Center Department of Pharmacology, Toxicology, and Experimental Therapeutics, contends that, while it is true that some tobacco smoke components are strong carcinogens in rodent mammary tissue, the resulting tumors do not reflect the histopathology or molecular alterations seen in the vast majority of human pre-malignancies or in primary tumors except for their estrogen dependency. “A case in point is that carcinogen-induced mammary tumors in rodents are diploid, and human ductal breast cancers are seventy to ninety percent aneuploid,” says Li. “Only breast tumors induced in rodents by estrogens are highly aneuploid. This would suggest why there is only a modest link between tobacco smoke and human breast cancer risk.”

Salmon and colleagues at the Cal/EPA OEHHA dispute the validity of Li’s observations. Salmon points to the differences not only between rodents and humans, but between different rodent species. “For instance,” he explains, “most mammary cancers in mice are hormone-independent at the time of detection, whereas nearly all rat mammary tumors are hormone-dependent initially. Human mammary tumors are intermediate in this regard, although the balance varies with age at diagnosis. However . . . both rats and mice are susceptible to mammary carcinogenesis by tobacco smoke constituents.” Salmon adds that karyotypic changes including aneuploidy are commonly seen at the later stages of tumor progression, but are not necessarily related to the initial cause of the tumor, being instead often dependent on host-related factors.

The data from epidemiological studies are even murkier. Taken as a whole, the 26 studies on the topic to date appear inconclusive, points out Alfredo Morabia, a professor of epidemiology at the City University of New York and lead author of a study in the 1 May 1996 issue of the *American Journal of Epidemiology* that did show a link. “There are large prospective studies that found no association, but some case–control studies find a strong association and others a weak association,” he says. “Some studies indicate that the issue is with younger women, others with older women.”

Others put it more bluntly. “If smoking was a major cause of breast cancer, we would have found it by now,” says Dale Sandler, chief of the NIEHS Epidemiology Branch, the researcher who published an article linking SHS exposure with several kinds of cancers in the January 1985 *American Journal of Epidemiology*. Li adds that one would anticipate that if carcinogen–DNA adducts were an important end point in human breast cancer, this would eventually translate into mutations in human breast cells. “This is . . . not the case in sporadic ductal breast cancer, which comprises greater than ninety percent of all breast cancer cases,” he says.

The researchers who are convinced that the data support a link between SHS and breast cancer counter that they do not claim that such exposure is the major cause of breast cancer, but simply one cause that many women can easily avoid. Mark Miller, a public health medical officer at the OEHHA, and Johnson were among the authors of *Proposed Identification of Environmental Tobacco Smoke as a Toxic Air Contaminant*, the Cal/EPA’s first publication to claim a causative link between exposure to SHS and breast cancer. The 528-page report was published in 2005 as part of the evidence mustered by the agency in its ultimately successful bid to become the first state to identify SHS as an air pollutant that could be regulated by the state. The peer-reviewed report devoted 56 pages to the toxicological and epidemiological evidence for a link between breast cancer and passive smoking, including a meta-analysis of the 19 studies available at the time. The WHO is in the process of republishing the report in other languages for worldwide dissemination.

Miller, Johnson, and colleagues also presented their case in a review article published in the February 2007 issue of *Preventive Medicine* with the goal of providing additional information about how they made their determination for the medical and public health communities. The new paper reiterates that women regularly exposed to SHS increase their relative risk of developing breast cancer by age 50 between 68% and 120%. These estimates were first calculated and published in a meta-analysis by Johnson that appeared in the 20 November 2005 issue of the *International Journal of Cancer*.

## Exposure Assessment and Age

There are two important reasons why the breast cancer risk from passive smoking can be difficult to tease out from earlier studies: exposure assessment and age. Melanie Marty, chief of the Air Toxicology and Epidemiology Branch of the OEHHA and a coauthor of the *Preventive Medicine* paper, explains, “Exposure assessment is always an issue in epidemiology studies, unless they are lucky and have lots of exposure data. In the case of secondhand tobacco smoke, many of these studies didn’t do a very thorough job of determining how long people were exposed, when in their lifetimes they were exposed, how much they were exposed.” Further, she continues, if exposure to SHS is not assessed carefully, “you are going to mix up the group that you think is unexposed with the group that you think is exposed.”

To address the issue of accurate exposure reporting, the 2005 Cal/EPA report identified a subgroup of studies that did a better job of assessing exposure, which were weighed more heavily in the final evaluation. The report also broke out younger, primarily premenopausal women—which it defined as under the age of 50—as the most vulnerable. This subgroup was first highlighted by Johnson in 2000, and the idea has been reaffirmed in recent findings. For instance, although researchers from the M.D. Anderson Cancer Center recently reported a dramatic 7% overall drop in breast cancer rates between 2002 and 2003, the decline was mainly observed in women aged 50 and older. The drop among younger women was much lower—only 1% for women aged 40 to 49, for example. These data were presented in December 2006 at the San Antonio Breast Cancer Symposium.

Only fourteen of the studies looking at passive smoking and breast cancer allowed analysis by menopausal status, according to the 2005 Cal/EPA report. These included ten case–control studies and four prospective cohort studies that began following a large group of women before any had the disease. Thirteen of these studies reported elevated risk estimates for breast cancer in premenopausal women, and the risk was statistically significant in seven of the studies.

The preponderance of case–control studies is a weakness of the case for linking breast cancer with passive smoking, an issue that Thun stressed in his debates with Johnson last summer. All things being equal, epidemiologists generally consider the findings of cohort studies to be more persuasive than those of case–control studies, because exposure information is ascertained before the development of disease and because both cases and noncases arise from the same study population. Beral points out that “every textbook of epidemiology says that once someone has a disease they might remember things differently.”

In response, Johnson says he doubts that recall bias explains premenopausal risk, pointing out that one would expect to observe similarly increased risk for pre- and postmenopausal women, which has not been seen. He says it is very difficult to collect good information about passive smoking in prospective study questionnaires.

“In a cohort study, you [might] have to interview a hundred thousand [people] in order to get a thousand cases. In a case–control study, you may have a thousand cases and a thousand controls, so you have to interview two thousand to find out the demographic and exposure information of interest,” he explains. “Because you have to interview fifty times as many people [in a cohort study], there’s a much higher price [in terms of administering the questionnaire] associated with every question you ask about exposure—the depth and quality of the exposure measures tend to be less unless it is a real focus of the cohort study.”

Further, says Russo, a woman might not know whether her grandparents, parents, and other relatives living in the same household smoked, and how much and for how long, nor whether her mother smoked during pregnancy.

Another issue that continues to plague efforts to link passive smoking to breast cancer is the fact that many researchers feel that epidemiological studies have not conclusively linked even active smoking to the disease. This, says Jonathan Samet, chairman of the Johns Hopkins Bloomberg School of Public Health and the senior scientific editor for the Surgeon General’s 2006 report, is one reason that report called the breast cancer–passive smoke evidence “suggestive but not sufficient.”

Stanton Glantz, a professor of medicine at the University of California, San Francisco, Medical School, claims this is because many of the most recent reports on active smoking weren’t considered when the Surgeon General’s report was being produced. The *Preventive Medicine* paper says that six large prospective studies have now found a statistically significant elevated risk for breast cancer among smokers for at least some metrics of exposure.

Peggy Reynolds, a cancer epidemiologist at the Northern California Cancer Center, is the lead author of one of the case–control studies that does support a link between active smoking and breast cancer, published in the 7 January 2004 *Journal of the National Cancer Institute*. She and her colleagues have been following 116,544 California teachers since 1995, and reported that the incidence of breast cancer among the cohort’s current smokers was higher than that for members who had never smoked. She said that the risk doubled for smokers with more than 31 pack-years—the equivalent of smoking one pack of cigarettes per day over the course of 31 years—compared with nonsmokers.

Although Reynolds did not report finding a link between passive smoking and breast cancer in that paper, she stresses that this could be because the questionnaire used to capture the data included information about whether women were exposed to smoking only in their homes. In a follow-up questionnaire, she and her colleagues asked more detailed questions to include other sources of passive exposure, particularly the workplace, and those data are currently being analyzed. These additional data are important because, although household exposures represented the major exposure source for women in this cohort during earlier decades, “following 1970, the workplace became women’s most important source of exposure to secondhand smoke,” she says.

In both their report and review article, Johnson and the Cal/EPA researchers also evaluated the biological plausibility of just how exposure to SHS might cause breast cancer, and concluded that “the chain of evidence indicates that a causal association is highly plausible.” Glantz points out that this isn’t always taken into consideration in epidemiological studies, which he argues gives the team’s conclusion all that much more weight.

## A Nonlinear Relationship

However, the chain of evidence regarding biological plausibility doesn’t fit neatly with the fact that active smoking does not cause a significantly higher number of breast cancers compared with passive smoking exposure. “One reasonable biological explanation for the similarity in risk would be that the tobacco smoke exposure pathways might become saturated at levels of exposure associated with regular secondhand smoke exposure, so that the higher exposure [from active smoking] would not further elevate the risk,” Johnson says.

It also appears that active smoking may partially mitigate effects of carcinogen exposure on the breast in smokers by reducing their estrogen levels. This fits with the M.D. Anderson study, which credited the declining popularity of hormone replacement therapy for the decreasing rates of breast cancer among older women. It might also explain why passive smoking, which is hypothesized to have less impact than active smoking on estrogen levels, could be associated with breast cancer. The problem with this, as Thun points out, is that no toxicological data exist to show there is a nonlinear relationship between the effects of low and high doses of smoke exposure.

Another potential explanation is that women may be especially susceptible during a key “window of exposure” that researchers have previously identified—namely, between puberty and when a woman bears her first child. Data from Hiroshima and Nagasaki, as well as from the treatment of young women with Hodgkin disease, show that the breast is not protected from potentially harmful environmental agents until it becomes fully differentiated in preparation for producing milk. This does not occur until the first full-term pregnancy. Pierre Band of Health Canada’s Division of Epidemiology and Cancer Prevention led a case–control study that supports the “window of exposure” hypothesis. Published in the 5 October 2002 issue of *The Lancet*, it showed that women who started smoking within five years of menarche were around 70% more likely to develop breast cancer than nonsmokers.

Many researchers interviewed for this article agreed that drinking alcoholic beverages does promote breast cancer, but not everyone felt that this factor has been adequately separated from tobacco exposure. “The breast cancer–alcohol association . . . is quite widely reported, but it is very hard to quantify and separate from the effects of concurrent active and passive smoking,” Salmon points out. “There’s also a complex multiway interaction between alcohol intake, smoking exposures, hormone levels, obesity, and breast cancer,” he adds.

The evidence for a breast cancer–alcohol link was presented in a large meta-analysis by Beral and colleagues that included over 50 studies and data from more than 150,000 women. That analysis, published in the 18 November 2002 *British Journal of Cancer*, found no link between active smoking and breast cancer, but Reynolds says her study showed otherwise; the data showed that the link between active smoking and breast cancer held whether or not alcohol consumption was considered. She says, “We did a second analysis . . . limited to nondrinkers, and we found the same elevated risk association.”

Johnson suggests that because passive smoking was not considered in Beral’s meta-analysis of alcohol risk, it is possible that it could have confounded the alcohol association results. He adds that the study simply compared women who said they currently smoked to women who reported smoking previously and those who said they never smoked, with no consideration of how much the women had smoked over their lifetimes.

Salmon says that if there is in fact an increase in breast cancer incidence directly caused by alcohol—as opposed to an association related to co-exposures—then promotional effects on cell growth might be involved. “At least,” he says, “there is a contrast with tobacco smoke, where genotoxic carcinogens are clearly present and involved along with other types of effects, and carcinogen–DNA adducts have been observed in the breast tissue of exposed women.”

However, Li notes there are numerous studies that show that women metabolize alcohol much more slowly than men, and that serum concentrations of 17β-estradiol are elevated after alcohol ingestion. “This,” he says, “is likely the reason why alcohol ingestion increases breast cancer risk.”

Although it had yet to be published when this article was being written, a new analysis by Beral and colleagues that linked alcohol to breast cancer is already having an effect on this debate. Thun said that after being briefed on the new data, he decided to hold off on moving forward with a workshop that the American Cancer Society planned to fund to identify the outstanding questions in the breast cancer–passive smoking debate. Beral would not comment on the data other than to say that the “findings are essentially null.” She said the analysis included 22 studies as well as new data and “several measures of exposure to secondhand smoke.” An article in the 1 January 2007 *Boston Globe* said the study focused on 1.3 million women aged 50 to 64, a different population than what the Cal/EPA scientists say is at greatest risk.

## What Next?

While some members of the research community are perturbed by the disagreement regarding the strength of the evidence connecting passive smoking and breast cancer, Samet says he thinks it is to be expected. “People should not be surprised if review groups don’t exactly come into complete alignment. Different groups bring expert judgment to bear in somewhat parallel but not definitely overlapping processes. I don’t think that the way we approach evidence review and synthesis is leading us astray,” he says.

Samet says that he would like to see the biological understanding advanced on ways that SHS could cause breast cancer. “Of course the simple story is that there are carcinogens in tobacco smoke, and they reach breast tissue, which is true,” he says. “[What makes the issue so complex is that] we would expect that far greater doses of these carcinogens would reach the breast tissue of women who actively smoke. We have not seen a clear signal showing that this is the case. I would like to see the biological framework laid out and better understood, as well as watch the epidemiological evidence grow.” Both the National Cancer Institute (NCI) and the NIEHS are funding research aimed at providing additional evidence on the topic. [For more information on this research, see “Centered on Breast Cancer,” p. A132 this issue.]

The Cal/EPA researchers contend it is likely that there are a number of subgroups genetically susceptible to breast cancer who could be especially sensitive to tobacco smoke exposure depending upon the polymorphisms of several genes. This is plausible, says Deborah Winn, acting associate director of the NCI’s Epidemiology and Genetics Research Program. “To actually look at gene–environment interactions and then try to look at breast cancer subgroups—you run out of numbers very quickly,” she says.

For that reason, researchers with the NCI Cancer Genetic Markers of Susceptibility initiative are doing genome-wide scans on more than 500,000 single-nucleotide polymorphisms and looking for changes between breast cancer patients and controls in a hypothesis-free approach. “Eventually, you might find things that map up to candidate genes that you already thought—on the basis of function or their role in estrogen metabolism—might be involved,” Winn says. Other research is focused on more detailed looks at promising candidate genes, she says.

Says Russo, “The fact that carcinogen-metabolizing enzymes such as CYP1A1 are increased in both lung and breast cancers, but that the death rate from lung cancer in American women has increased six hundred percent from 1930 to 1997, whereas breast cancer has remained stationary during the same period, might suggest that women carriers of susceptibility genes would be more prone to develop both breast and lung cancer. Unfortunately, statistics on the incidence of breast lesions in women diagnosed with lung cancer are almost nonexistent.”

Sandler is currently in the process of recruiting women for an NIEHS-funded prospective study of sisters of women who have or had breast cancer; this group is twice as likely as other women to develop breast cancer. She has already enrolled 30,000 such women who don’t have breast cancer, and she aims to sign up 20,000 more. “The purpose is to look at environmental and genetic risk factors for breast cancer—certainly . . . we’ll be looking at their life history of exposure to cigarette smoke, their own and through their parents and their spouses and their roommates and their jobs,” she says. “The questionnaires were designed to do a thorough job of looking at it.”

Just what all this research will show is unclear. The only certainty, Samet says, is that “scientific evidence will continue to accumulate on this topic.”

## Figures and Tables

**Figure:**
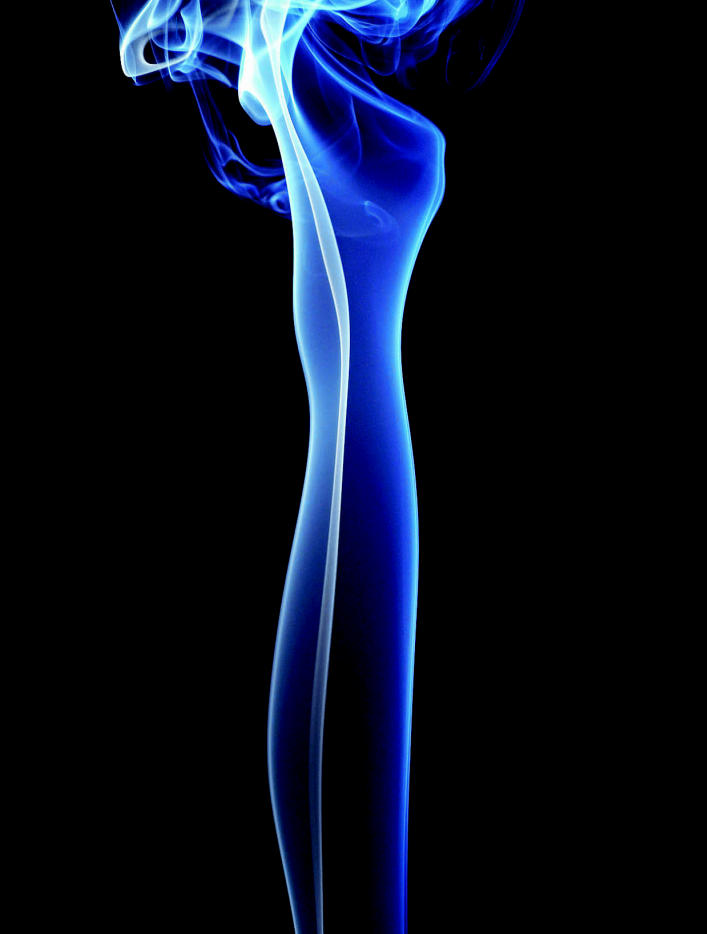


**Figure:**
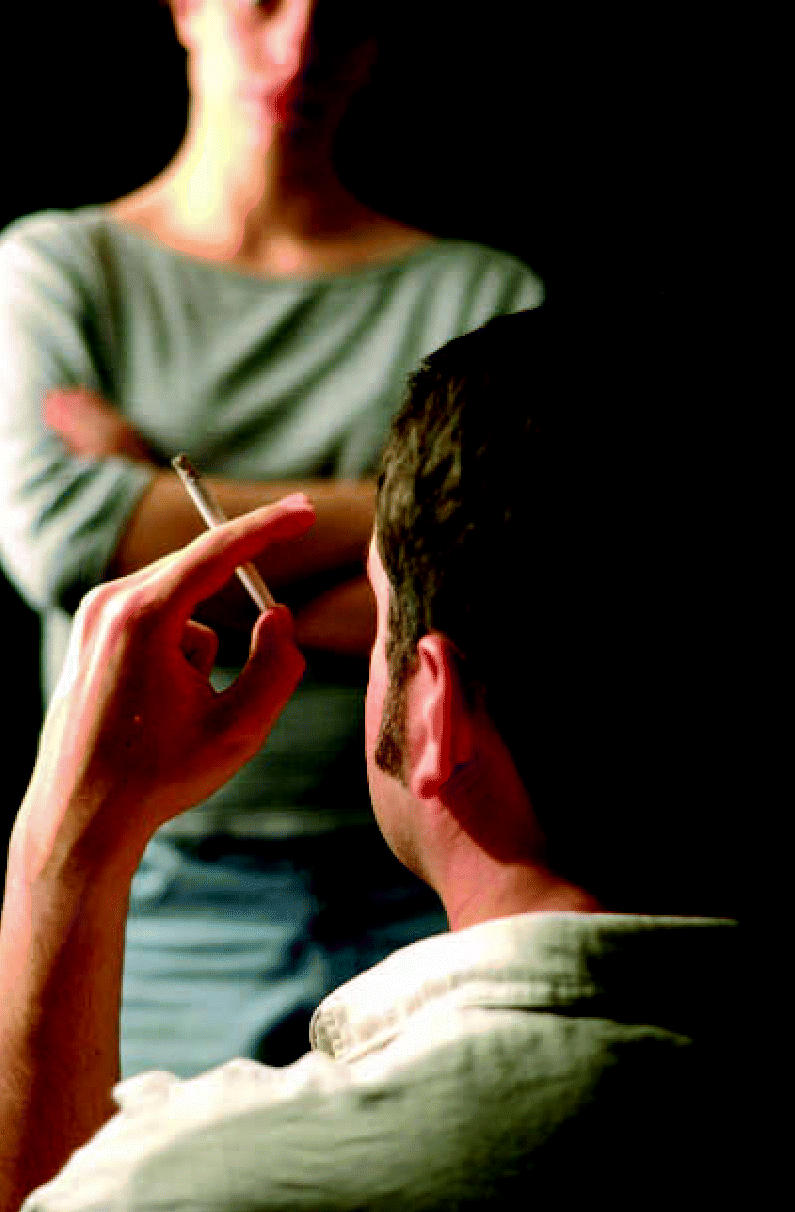


**Figure:**
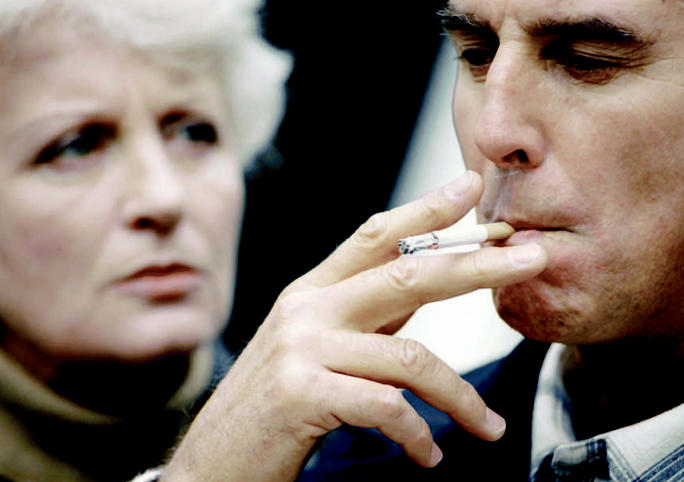
A matter of age Much of the discrepancy in findings related to passive smoking and breast cancer may relate to differences between younger and older women. Premenopausal women are believed to be more vulnerable to breast cancer.

**Figure:**
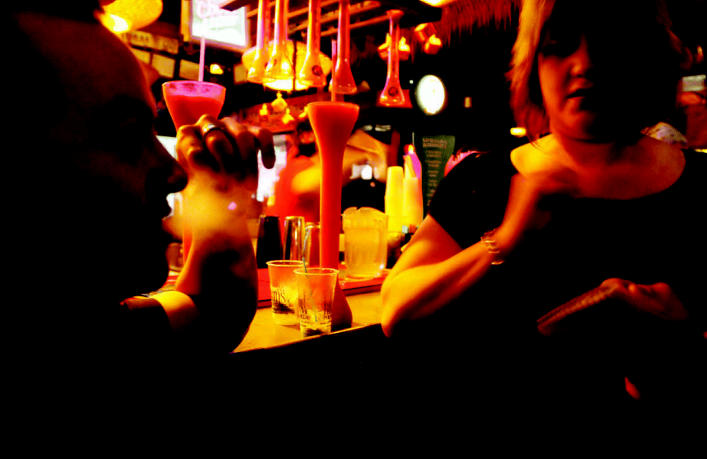


**Figure:**
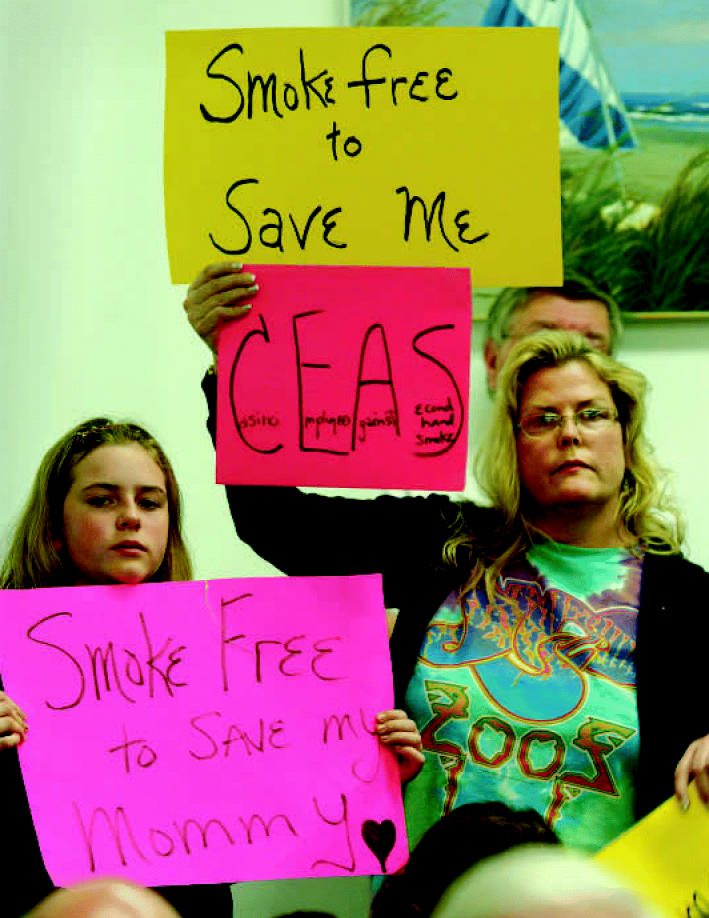
A continuing revolution? As the female workforce grew at the end of the twentieth century, the job site became an important source of SHS exposure for many women. Today, however, health concerns and protests (like this one, right, at a January 2007 Atlantic City council meeting) have led to laws that protect such workers.

**Figure:**
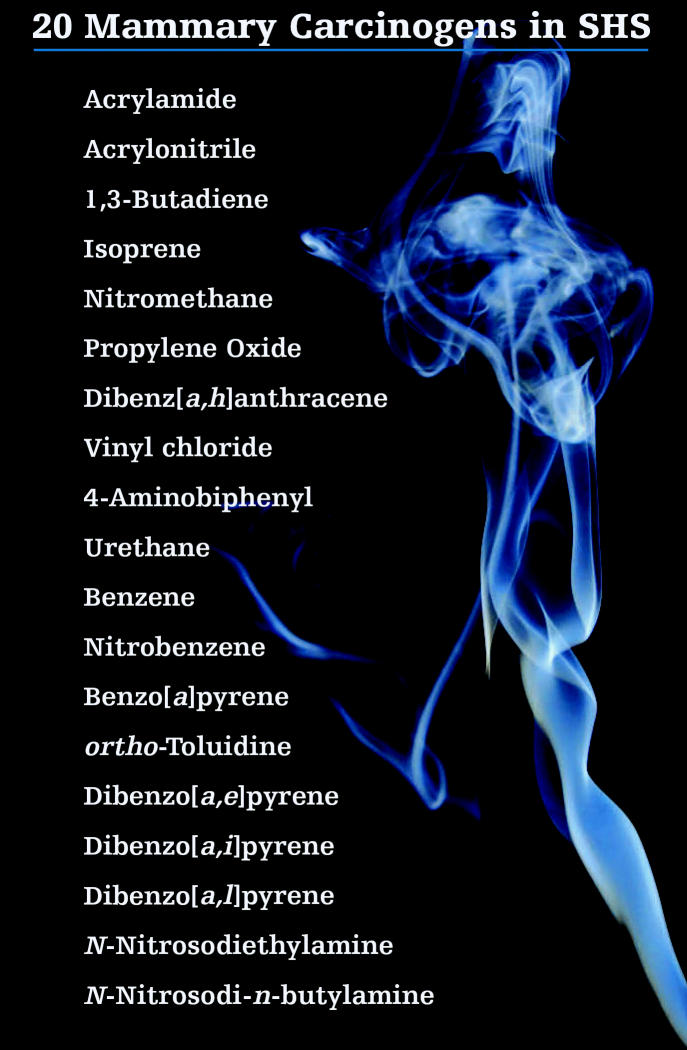
**Source:** Miller et al. Prev Med 44:93–106 (2007).

**Figure:**
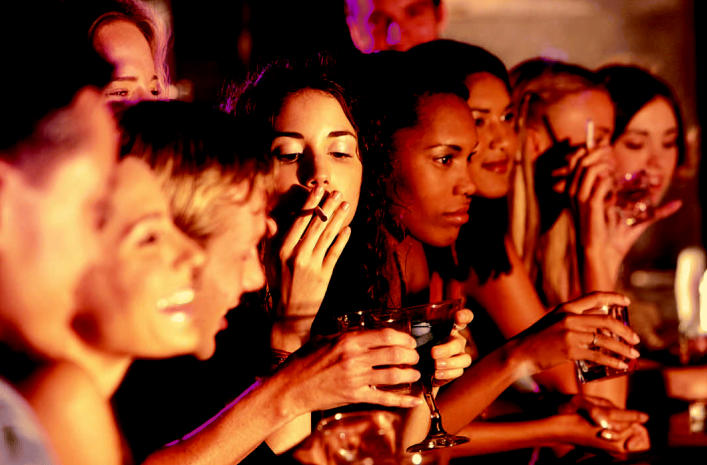
Noisy environment Drinking adds another dimension to the uncertainty about the effects of SHS. Breast cancer studies published to date have been unable to tease out the effects of concurrent active and passive smoking from alcohol intake.

